# Parents' experiences of collaboration between welfare professionals regarding children with anxiety or depression - an explorative study

**DOI:** 10.5334/ijic.986

**Published:** 2013-10-31

**Authors:** Catharina Widmark, Christer Sandahl, Katarina Piuva, David Bergman

**Affiliations:** Karolinska Institutet, Medical Management Centre, SE-171 77, Stockholm, Sweden.; Karolinska Institutet, Medical Management Centre, SE-171 77, Stockholm, Sweden.; Department of Social Work, Stockholm University, SE-106 91, Stockholm, Sweden.; Karolinska Institutet, Medical Management Centre, SE-171 77, Stockholm, Sweden.

**Keywords:** patients' perceptions, collaboration, child and adolescent mental health, child welfare, holding environment

## Abstract

**Introduction:**

Well-functioning collaboration between professionals in the welfare sector has a strong influence on the contacts with parents of children and adolescents suffering from mental illness, and it is a precondition for the availability of support for these parents. This paper describes how such parents perceive collaboration between professionals in mental health care, social services, and schools.

**Methods:**

This was a small-scale qualitative study. Data were collected by in-depth interviews with seven parents of children and adolescents diagnosed with anxiety and depression. The families were selected from the Child and Adolescent Mental Health patient records kept by the Stockholm County Council (Sweden), and they all lived in a catchment area for Child and Adolescent Mental Health outpatient services in Stockholm.

**Results and discussion:**

Our results suggest that when the encounter between parents and professionals is characterised by structure and trust, it is supportive and serves as a holding environment. Parents think that communication links and coordination between professionals from different organisations are needed in the collaboration, along with appropriately scheduled and well-performed joint meetings to create structure in the parent-professional encounter. Parents also think that establishment of trust in this interaction is promoted by individual professionals who are available, provide the parents with adequate information, are skilled, and show empathy and commitment.

## Introduction

In Sweden, most of the public welfare agencies that deal with mental health care of children and adolescents are specialised. This situation creates a need for collaboration, which is a challenging task for the organisations and the professionals involved. ‘Collaboration’ can be understood in disparate ways depending on whether the professionals belong to the same or different welfare organisations. Another significant aspect is whether the professionals collaborate in integrated teams, in which they work side by side, or by participating in joint meetings when necessary, or through various other means of contact. The present study focused on collaboration between professionals from different Swedish welfare organisations (Child and Adolescent Mental Health Care, social services, and schools) which is achieved by joint meetings and other forms of contact. Previous research in this context shows a number of factors that have an impact on whether such interaction will be successful. Some of these factors concern organisations that share responsibility for management [1], and that collaboration is of common interest and also has a common goal [2,3]. Furthermore, it is relevant for professionals to have knowledge of each other's organisations and whether there is trust between themselves [4]. To have a clear understanding of the factors that promote or impede collaboration, it is important to achieve a comprehensive approach in the joint work [5]. In other words, there is a lot of knowledge concerning collaboration between professionals from different organisations and how these individuals perceive the interactions between them. It may also be of interest to explore how the families perceive the collaboration between the professionals, particularly considering that parents are strongly affected by a situation in which they are seeking support for their child and their family [6,7].

From a family perspective, it appears that a significant role is played by the encounter between the family as a system and the system comprising collaborating professionals from different organisations [8,9]. Unfortunately, it is difficult to confirm or refute our present knowledge in this area, because we have only a limited understanding of parents' perceptions of collaboration between the various organisations involved. Therefore, this study was undertaken with the aim to explore how parents of children and adolescents with mental illness view such interaction.

### Collaboration as support to the families

The availability of support for parents of children and adolescents with mental illness probably influences the children's recovery [10]. A literature review of international evaluations of such cases has shown that giving parents support significantly improved the mental health of the affected children [10]. One of the reasons those efforts were successful was that they were structured, that is, they followed a prearranged plan. When families with multiple needs have to seek support from several organisations in the welfare sector, it is likely necessary for those organisations to collaborate. These families often fall between the cracks, which emphasises the importance of adopting a comprehensive perspective [5,11,12]. Well-structured collaboration between the organisations involved is needed and probably also serves to support these families.

Structure in collaboration between professionals from different organisations seems to be an essential aspect when parents seek help for their child. The child's condition may have affected family functioning [13], and it is likely that the professionals will need structure in their contacts with one another to be able to help the family regain normal functioning. ‘Structure’ in this context is regarded as an activity that has an impact on human processes, either by making them clear and predictable (as in the contact between families and professionals), or by improving their functioning (as in the interactions between professionals from different organisations).

The structure of the contacts between collaborating professionals from different organisations appears to be connected with the interactive process between the service users and the professionals. Ahgren et al. [9] obtained evidence that adequate structure of integration (regarding professional skills and competence, information, and availability) between different care providers is a prerequisite for development of the interpersonal and interprofessional process (regarding trust and responsibility between the provider and the user, and different professions working together), which in turn influences the outcome for the adult service users. Spragins and Lorenzetti [8] conducted a literature review and found that patients' and caregivers' experiences of integrated health care highlighted the need for care coordinators and care plans that could be beneficial to both the users and the providers of such services. Care coordination was also experienced as being associated with the degree to which health care professionals communicate and consult with each other and with the patients. Care plans should be developed together with patients and shared among care providers [8,12,14]. Using the term ‘coordination’, we refer to support and treatment that must be coordinated in time, when there is simultaneous involvement of resources and professionals from different organisations. These professionals also have to coordinate their contacts with the family.

### Joint meetings

Joint meetings between professionals from different organisations and service users/families represent a common model for collaboration, and they are intended to provide the opportunity to discuss difficulties and appropriate treatment. Willumsen and Skivenes [15] described how four criteria based on a ‘deliberative model’ were used at review group meetings concerning children in residential care. One criterion stated that allowance must be made for differences in the participants' abilities to present their views and to deliberate. Accordingly, these authors discussed what they called ‘the deliberative forum’ as a strategy that can aid understanding through communication and thereby offer the participants a potential for learning. Forkby [16] described three categories of joint meetings that were referred to as network meetings: the full-scale intervention, structured network meetings, and open dialogue. This investigator explored network meetings as full-scale interventions and considered democracy and empowerment as strong ideological components of the meeting, and he also called for an ethical awareness on the part of the professionals. The joint meetings mentioned in our study correspond primarily to the second category mentioned by Forkby [16], that is, structured network meetings. These meetings include family members and professionals from the different organisations from which the family needs support, and they are held to allow the participants to discuss and to coordinate support and treatment.

### Key factors in collaboration between family and professionals

Previous research has often focused on the experiences of adult patients or service users, frequently highlighting the interactive process between the service users and individual professionals. In a study by Stanley et al. [17], it was noted that mothers whose children had been the subject of a case conference regarding child protection mentioned the availability of the professionals, which was perceived as being correlated with trust. Those mothers also described the most helpful professionals as ‘being there for me’, and this expression included availability, listening, and commitment to the service user, which in turn created trust. Beresford et al. [11] pointed out that service users viewed the interaction process as inseparable from the outcome, indicating the roles played by involvement, respectful treatment, and appropriate information. In the same way as seen between members of a family [6,7], it seems that parents and professionals develop interdependent patterns. Dumbril [18] explored the ways in which parents experienced and negotiated a child protection intervention, and found that the parents viewed the outcomes of treatment as being a result of the interaction between the professional and parental power. The parents who felt that professionals' power was being wielded over them tended to fight or play the game, whereas the parents who experienced that such power was being used on their behalf tended to cooperate with the intervention. Gross and Goldin [14] also identified power as a balancing factor in the liaison between the family and the team in an inpatient psychiatric unit, and they emphasised that it is necessary for the professionals to reduce the imbalance in this respect in order to be able to share with the family any decisions about treatment. Moreover, Ekwall et al. [19] reported that it is important for female patients to have the opportunity to influence their encounters with the professionals.

### Parents' perceptions of collaboration

In summary, some research has outlined the prerequisites for successful collaboration between different welfare organisations. The literature also provided information regarding the contacts between families and professionals in this context. However, as far as we can discern, there is a lack of knowledge about parents' perceptions of collaboration between professionals providing services in the different organisations. Thus, the aim of the present study was to gain a better understanding of the aspects of the professionals' collaboration that might be experienced as either successful and supportive or unsuccessful by the families with children affected by mental illness.

## Methods

### Data selection

Parents of children with a mental illness were invited to participate in individual in-depth interviews. It was of interest to assess the parents' views, because they are usually responsible for communicating with the professionals in different organisations about aspects such as pertinent planning for and treatment of their children in attempts to coordinate the various dimensions of the situation. The children, at least the adolescents, are no doubt also included in the planning and treatment, but they are probably not included in the parents' efforts related to collaboration between professionals from different organisations. For the present analysis, we selected suitable families from the Child and Adolescent Mental Health patient records kept by the Stockholm County Council (Sweden), focusing on children and adolescents with either of the two most common diagnoses: anxiety and depression. Another inclusion criterion was parental participation in at least one joint meeting together with professionals from at least two of the collaborating organisations (i.e. mental health care, social services, and schools). All families lived in a catchment area for Child and Adolescent Mental Health outpatient care in the southern part of the city of Stockholm. In Sweden, families seek contact with Child and Adolescent Mental Health on a voluntary basis. The staff at each outpatient clinic comprises counsellors, psychologists, and child psychiatrists with psychotherapeutic skills. In some instances, social services and schools try to motivate families to contact Child and Adolescent Mental Health based on their concern for a child. Furthermore, CAMH and the schools can be legally obligated to inform the social services if a child is being mistreated, and the Child and Adolescent Mental Health and social services and schools often collaborate in such cases. In addition, authorities in Sweden have a statutory duty to collaborate in the handling of cases involving maltreatment of children and adolescents [20].

Our study was approved by the Ethical Review Board of Karolinska Institute. A letter including a description of the project, information about confidentiality, and an invitation to participate in an interview was sent to the parents, and those who chose to take part signed consent in connection with the interview. Five parents were interviewed in the first phase of the study, and there were similar themes coming up. Two additional interviews confirmed these initial themes, and we considered the material to be saturated. Each interview lasted approximately one hour [21,22]. All but one of the interviews were held in the families' homes during the spring and early autumn of 2011, and they were conducted individually with one parent from each family without interference from other family members. In all, two fathers and five mothers were interviewed; four of them lived as a single parent and three in a conventional family. All informants were socially established with permanent work and housing, although one was on long-term sick leave. The seven children had a mean age of 14.2 years (range 9–16 years), and three were girls and four boys. At the time of the individual interviews, most of the children had been in the Child and Adolescent Mental Health records for 5 to 13 months and one of them just over 6.5 years. The contact was still ongoing for all of them. All interviews were conducted by the first author (C.W.).

### Individual interviews

Individual in-depth interviews were conducted using open-ended questions that focused on a few issues regarding collaboration between Child and Adolescent Mental Health, social services, and schools [23]. Initially, the parents were asked to describe their experiences at joint meetings with professionals in mental health care, social services, and/or schools. However, we recognised their responses as descriptions of their own views regarding collaboration, and hence, we chose to use the term ‘perception’. There were also questions concerning availability of the professionals, parental involvement in care planning, and whether the joint meetings had resulted in further efforts to improve the affected child's condition. During the interviews, additional questions emerged concerning treatment interventions, whether the treatment had led to improvement, and if the needs of the parents had also become visible to the professionals.

## Analysis

The data from the seven interviews were audio-recorded and then transcribed by a professional writing agency. The transcripts were subjected to conventional content analysis [24] using an inductive approach [21,22]. Inductive content analysis is recommended when knowledge is fragmented or when no previous studies have focused on the phenomenon of interest [25]. Based on previous research, we are aware of a number of factors that have an impact on the success of collaboration [1–3,5]. From a family perspective, it also appears that collaboration is of significance, but there is little research on that particular topic to contradict or confirm what is known at present.

The transcripts from all seven interviews were read at least twice and encoded using Nvivo9 ™, a computer program designed for coding text content. The purpose of this approach is to facilitate the drafting of codes and to organise the material. This entails manual operations, and helps to reduce the amount of work that has to be done at intervals and to avoid going back. Meaning units were extracted from each interview and encoded. In all, 261 codes were found and, based on these, the analysis resulted in two main categories and nine subcategories.

**Figure 1.**
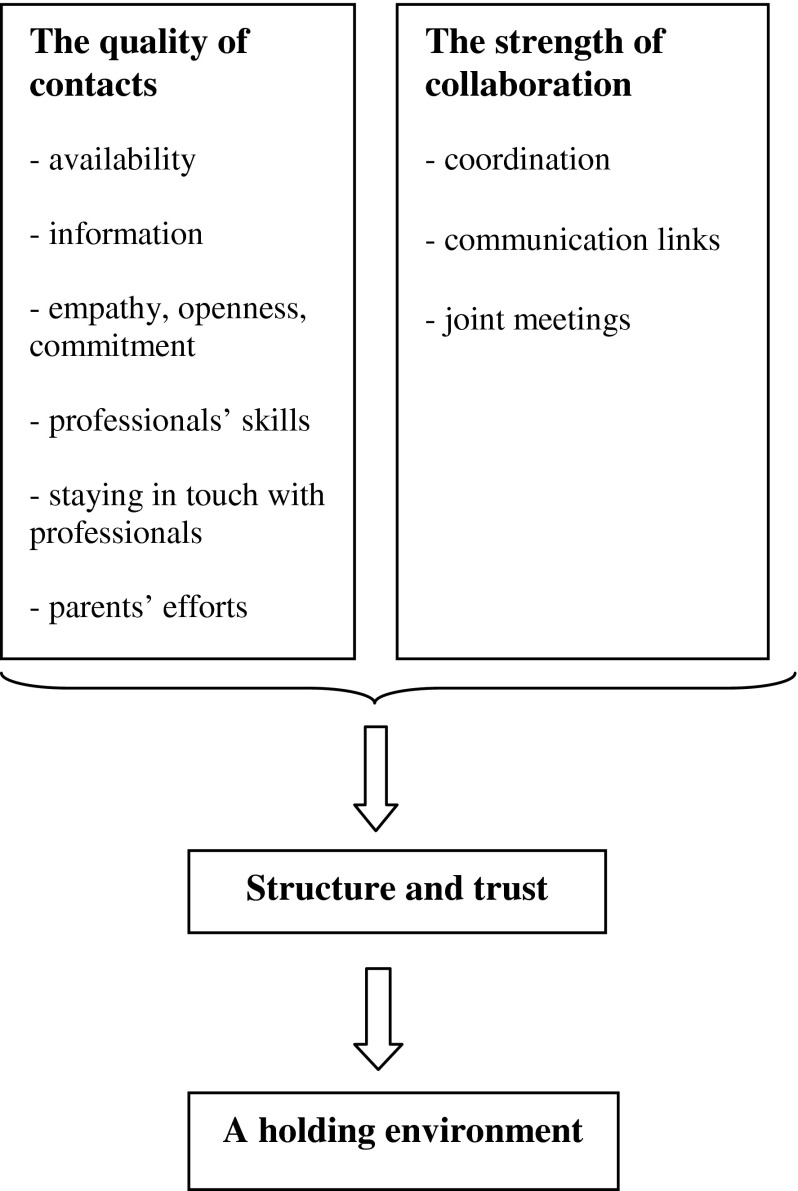
A tentative model of parents' perceptions of collaboration between mental health care, social services and schools.

The first author (C.W.) discussed and analyzed codes and themes together with the other authors. Discussions continued until consensus was achieved concerning the interpretation of the results, and the model was subsequently elaborated according to the consensus (Figure 1). This was done to neutralise potential bias and to validate the results [26], because the first author (C.W.) also had previously held a position as a unit manager of a Child and Adolescent Mental Health outpatient clinic in Stockholm.

## Findings

The interviews were focused on the parents' perceptions of collaboration between professionals in mental healthcare, social services, and schools, but the compiled data had a wider range than that. The parents also described their family situation with respect to how they felt about having a child who showed signs of mental illness, as well as their experiences of contacts with individual professionals. These aspects were important to help the researchers understand the parents' perceptions of collaboration between professionals.

The analysis resulted in two categories and nine subcategories. The two categories were labelled (1) the quality of contact and (2) the strength of collaboration**. The first of these included the following: the parents' encounters with individual professionals; the availability of the professionals; the information the parents were given; and how the parents were affected by the empathy, commitment, and openness of the professionals; the parents' attempts to seek help and their experiences of having numerous contacts with many different professionals; the parents' views on their own efforts to contribute to well-functioning collaboration with the professionals. The category designated the strength of collaboration comprised the perception of inter-professional interaction, the significance of coordination and communication links between the professionals, and the parents' perceptions of joint meetings with professionals from different organisations. The categories and subcategories and examples of codes are presented in Table 1.

A number of quotations are presented below to illustrate and facilitate comprehension of the categories and subcategories that were identified.

### Category 1: the quality of contact

#### Availability

Once contact had been established with the professionals, the parents experienced the availability of these care and support providers in different ways as the cases progressed. Trust was created when the professionals replied within a reasonable amount of time after they had been contacted by the parents, but there were also occasions when the parents had to wait quite a while before receiving a response.

She always calls me back. She might not call the same day, but she always calls. She makes you feel like you /…/ can trust her, and that's very important. (single mother)

#### Information

The parents expected to be given information concerning aspects such as treatment options, the various roles of the different professionals, and notification in advance if a meeting was cancelled. They felt there were deficiencies in this context, and some of them had ideas about how to ensure that the information would reach them.

Above all, they should sit down, and they should have prepared [for this] by finding all /…/ kinds of options that might exist and then present them in different ways. (single father)

#### Empathy, openness and commitment

In each contact that occurred, the parents perceived that it was highly important for the professionals to be able to display their empathy and commitment, and also to show whether or not they recognised the parents' problems. In the eyes of the parents, the professionals conveyed their commitment by not letting go and instead doing follow-ups. They were perceived as open and positive when they listened to the views of the parents and the children.

Well, this thing about not letting go…You feel like no one has let go of us, we're not just out there all on our own, just the opposite, we've got follow-up, and you could always call if that was needed…(mother in family)

When the parents perceived a lack of empathy and commitment on the part of a professional, it was difficult for them to know whether the professional actually understood the family's situation. If a professional (in an ongoing contact) had not initiated interaction with the parents to further help the family, the parents often said ‘nothing happened’, which meant that they had to wait and in the meantime, no support was offered.

…nothing happened until in November, when I got the school going about how we needed to talk about this, ‘How can we kind of get her to go back to school?’ (mother in family)

When professionals used terminology that the parents were not very familiar with, a gap was created between them. The parents also experienced closedness in the interaction when they felt that professionals did not listen to what they had to say.

…it's that kind of term, I don't know what it's called /…/ and they used [terms] that we ordinary people don't understand over and over again…

(single father)

#### Professionals' skills

Some parents noted differences between the professionals they met, and they thought these disparities influenced the treatment their children were offered. They observed how the professionals applied their skills and experience to be able to make their assessments, emphasising that in some cases, they were lucky, and sometimes not. It seemed that parents who regarded the professionals as competent and experienced felt secure, and their own disorientation abated. On the other hand, some parents had interactions with professionals who did not behave as expected.

…so if there's anything I've read between the lines, its maybe that we've been lucky because we've had this team in this particular school, that they've done their job very well and they've really wanted it to work (mother in family)

#### Staying in touch with professionals

From the beginning, the parents were in contact with professionals at the Child and Adolescent Mental Health services and the schools, and eventually also with staff at the social services, and they participated in a wide variety of meetings. It was confusing and difficult for them to coordinate these activities. They often had to tell their story over and over again to different professionals. Most of the parents also had jobs to manage, and it was exhausting for them to have to handle this double workload.

…I was so darn tired of having to make so many calls and keep doing this. ‘You'll have to pay me /…/ I can't manage my job and keep this up [at the same time]’. (single mother)

#### Parents' efforts

Most of the parents expressed that it was essential to have a positive attitude towards the professionals. They meant that, as a parent, it was necessary to show willingness to cooperate, and that being accusatory when something went wrong or was unclear was not a viable approach.

It also has a little [to do] with one's own attitude /…/, we're not trying to accuse anyone or put the blame on anyone, or demand that someone else solves [the problem], but rather we have to do it together, [so that] everyone feels like………they've done their part. (mother in family)

The parents indicated that their openness towards the professionals was just as significant as the other way around. It was important to them to be honest about their need for help. However, the parents also pointed out that there were limits, for example, they felt it was necessary to speak up when treatment was not helping their child or when a professional acted inappropriately.

‘But no, you jump on me as a parent /…/ and say that I'm making a mistake, and then I have to wonder where you got your information.’ After that day she really understood that you can't treat me like that anyway. (mother in family)

### Category 2: the strength of collaboration

#### Coordination

When a difficult situation arose, the parents felt a need for relief, and it seemed that the most important requirement in that context concerned coordination among the professionals. The parents regarded it as nearly a full-time job trying to maintain contact with various organisations and professionals. Some of them described how they had to fulfil two roles, one as a mother or father and the other as a coordinator between professionals, and they also related that receiving help from a professional coordinator could be reassuring and enable them to focus on being parents.

…and then they have a coordinator who handles all these aspects. I don't have to be more than a mother /…/ It's much, much easier now.

(mother in family)

#### Communication links

According to the parents, collaboration would proceed in a smoother fashion if the professionals had communication links with each other (with the parents' consent). The parents would be relieved if the professionals informed each other about the current situations in the families and tried to reach a common understanding of the children's difficulties. Communication links would also give the professionals the opportunity to consult with each other, which would even benefit the parents.

No, I hope that it will be a little smoother and easier in the future, maybe that they have some of their own contact channels when they want to get a hold of each other and such, with the parents' consent that is. (mother in famiy)

#### Joint meetings

When a joint meeting was proposed by either the parents or the professionals, it was planned as a forum for joint discussion of the situation at hand and to find appropriate strategies to alleviate the difficulties. At such a meeting, professionals from various activities and organisations took turns describing their views on the child's problems, which also included the opinions of the parents. Carefully planned and well-performed joint meetings lessened the burden on the parents and were experienced as having a positive effect. The parents also seemed to think that joint meetings had given the professionals new ideas.

When you left [the meeting], you felt a little relieved and thought that now there's something to work on, we're going to do everything, from many different places…(mother in family)

There were also negative experiences of joint meetings. A parent could have feelings of being cornered. Also, in some cases, structure was lacking or vague with regard to scheduling of a meeting, and participating professionals were occasionally delayed. The parents regarded that as unsatisfactory and at times ambiguous. Moreover, they complained about absence of continuity in professionals' participation from one meeting to another, because that often meant that the professionals were not up to date on the situation of the family in question.

…with each passing meeting, they sent new people, and they never knew anything, because they hadn't been at the previous meeting…

(single mother)

### Summary of the results

We were interested in obtaining a picture of how parents seeking help for children and adolescents with mental illness perceive collaboration between professionals working in different organisations in the welfare sector. Our results are summarised in two categories: the quality of contact and the strength of collaboration.

The first category (the quality of contact) illustrates the importance of the professionals' availability to the parents. Being given inadequate information about aspects such as treatment alternatives made it difficult for the parents to make their own judgments when necessary. The professionals' ability to show empathy and commitment gave the parents an idea of whether these care providers actually understood the family's situation. The parents noted the professionals' skills and associated them with the treatment that was offered. When the parents decided to seek help, most of them initially had to maintain contact with many professionals in different organisations at the same time, and it was confusing and exhausting to have to try to keep all these elements coordinated. The parents considered that their own efforts contributed to well-functioning collaboration. Being accusatory was not a viable approach.

The second category (the strength of collaboration) concerns the parents' perception of interactions between professionals from different organisations. The parents called for coordination and communication links between the professionals and felt relieved when such interconnections existed. Furthermore, they appreciated it when joint meetings with professionals from different organisations were well prepared and properly scheduled. If the opposite was true, such meetings were regarded as unsatisfactory and in some cases vague, and there was also a likelihood that the parents could feel cornered during a session.

## Discussion

Considering the first aspect of the encounters (i.e. trust), it emerged that the parents were affected by their experiences of the availability of the professionals. This seemed to have a deeper meaning than merely being available by phone, because it also included the professionals' tendency to listen, to be open, and to show commitment to the parents. The professionals' ability to display empathy and commitment was perceived as indicating recognition and understanding of the family situation, which, from the perspective of the current families, was of the greatest significance but was not always achieved. In this regard, the expression ‘being there for me’ that was described by Stanley et al. [17] indicates a way for parents to summarise these needs. The cited authors highlighted the importance of service users being able to trust professionals and that this is partly connected with the availability of these care and support providers.

Yet another issue in this respect concerned the information that was given. The parents in our study indicated that they required adequate information to be able to orientate themselves in relation to illnesses and treatment. Moreover, they felt that being given appropriate information would enable them to be involved in the planning of measures and decisions affecting their children's health, which Chan [12] and Spragins and Lorenzetti [8] have also identified as essential. Beresford et al. [11] referred to the interactive process between the service user and the professional as including a perception of being involved and being given relevant information, which also affects how an outcome is experienced. Furthermore, Ahgren et al. [9] have stated that the availability of relevant information for the service users and the professionals' capacities and abilities are manifestations of the structure of integration between professionals from different organisations.

Regarding the second aspect of the encounters (i.e. structure), we found that the parents considered it extremely important to have access to a professional coordinator. They initially found themselves in a situation in which they had to play dual roles as coordinators for the professionals and as parents, and this was too difficult and sometimes confusing for them. They were also apprehensive about the professionals' knowledge of their families' current situations and expected these care providers to communicate with each other about the cases. Such collaboration would relieve the parents of the necessity of constantly having to provide the professionals with relevant information. By expressing this, the parents emphasised the need for interprofessional and interpersonal communication links. In other words, there was a need for structure in the interaction in two directions: on the one hand, between the professionals and the parents, and on the other hand, between the professionals from different organisations. Spragins and Lorenzetti [8] also stressed this aspect and suggested that the communication should be organised by coordinators or case managers.

The parents in our study conveyed an obvious need for structure when they described their participation in joint meetings with professionals from different organisations. Similar to Ekwall et al. [19], we concluded that the parents in our study were concerned about having the opportunity to influence interactions with the professionals. The parents felt that everyone listened to them when they took part in appropriately scheduled and well-performed meetings in which all the participants had the opportunity to describe their views on the situation. This also made them feel involved in discussions about matters such as the treatment offered to their children, which was most important to them. Unfortunately, these meetings were not always successful. Gross and Goldin [14] have indicated that the ability to perform a well-functioning network meeting might be regarded as the result of the professionals' ability to balance the power between themselves and the parents. This agrees with Forkby [16], who suggested the importance of an ethical aspect. This manner of balancing the power probably made it possible for the parents in our study to contemplate their own contributions to their interactions with the professionals. It is plausible that the parents were still ambivalent in their attitudes, as discussed by Dumbril [18], but they considered cooperation to be a preferable approach that was likely to improve their children's conditions. Finally, some parents regarded joint meetings as an opportunity for professionals to learn from each other. This has also been suggested by Willumsen and Skivenes [15], who pointed out that the potential for learning applies to all the participants in joint meetings.

Our results revealed two essential aspects of the encounters between parents and professionals that could render these interactions supportive. The first aspect concerned trust and included the various components of the perceptions of the process of individual professionals interacting with the parents (the quality of contact) [11,17]. The second aspect concerned structure and was related to perceptions of interprofessional interaction (the strength of collaboration) [8,9]. The professionals who were able to establish trust in their encounters with the parents and structure in the collaboration between professionals from different organisations created a holding environment for the parents. Such an environment is defined by Petriglieri and Petriglieri [27, p. 50] as *a social context that reduces disturbing affects and facilitates sense making*. We understand this as a context in which the professionals create a space or an arena where their encounters with the families can be as supportive as possible. Figure 1 summarises our results identifying factors that influence encounters between the parents and professionals.

Like Ahgren et al. [9], we found that structure of integration in collaboration between professionals from different organisations had an impact on the interaction between the professionals and the parents, and this structure was manifested in terms of coordination, communication links, and well-conducted joint meetings. However, there may also be barriers to collaboration between professionals in health care, social services, and schools, which, as has previously been shown [28], can be created by deficiencies in clarity with respect to allocation of responsibilities, confidence between professionals, and the professionals' encounters. These results suggested the significance of a holding environment [27] on the managerial side to benefit the professionals in their joint efforts to develop collaboration into a long-term and sustainable process. In this context, we refer to a management function that is ‘holding’, because it involves being committed to and responsible for collaboration and performing follow-ups concerning collaboration on different levels between the different interacting organisations. In the current investigation, we found that creating well-structured collaboration in combination with trust in individual professionals represented a way to offer the parents support, as has also been observed by Spragins and Lorenzetti [8] and Beresford et al. [11]. In addition, this requirement for structure and trust might be looked upon as a holding environment that is provided by the professionals to facilitate encounters with the parents when different welfare organisations are involved. Thus, these creative efforts are aimed at achieving two such environments—one including the professionals and the families, and another including the professionals and management—which exist in parallel and in that sense are interconnected, as has also been described by Ahgren [9]. Consequently, in future research, it might be beneficial to use a perspective that considers collaboration as parallel processes in ongoing communication between the service users and the professionals at various levels. In future research, it might also be beneficial to the treatment outcome to ascertain whether that aspect is affected by a holding environment.

It is also plausible that our results can have practical implications related to the need for coordination in order to create the type of holding environment indicated here. In individual cases, the participating professionals from different organisations might intentionally appoint a coordinator to benefit the families that have multiple needs related to children's mental illness. On a managerial level, it might be appropriate to have a coordinator who is responsible for collaboration between the organisations that provide support to affected children and their families. Indeed, these are interesting aspects that could be a basis for future research.

## Limitations

Our work was conducted as an exploratory, small-scale qualitative study, and thus, it had certain limitations. We used two criteria for data selection. First, we considered it appropriate to choose children with anxiety and depression registered as their main diagnoses, because these conditions predominate in the Child and Adolescent Mental Health records kept by the Stockholm County Council. Second, it was decided that the parents were to have participated in at least one joint meeting with professionals from at least two of the organisations of interest (county council, municipality, and schools). We found that fewer joint meetings than expected had been held concerning the mentioned diagnoses. However, we have no reason to believe that the patients we selected for our study differed from other categories of patients in terms of collaboration. Instead, we chose the most common diagnoses in order to adequately represent the group of patients in the Child and Adolescent Mental Health records, that is, to strengthen the reliability of our results.

The parents were interviewed in two phases. First, five parents were interviewed, and similar themes emerged. Thereafter, two additional interviews confirmed the initially identified themes, and thus, we considered the data to be saturated. This demonstrated that the number of interviewees in our assessment was sufficient to provide reliable results.

Another possible limitation of our study was that the first author (C.W.) had previously worked as a unit manager of Child and Adolescent Mental Health outpatient care run by the Stockholm County Council, which might have influenced her interpretations and conclusions regarding the data. However, we validated the results by continuous consensus discussions including all the authors, which resulted in the themes and categories described in this paper.

## Conclusions

In the present study, the parents of children suffering from depression or anxiety felt that they were profoundly affected by the encounter between the family as a system and the collaborative system of professionals. They described the need for coordination and communication links to promote collaboration between the professionals, along with appropriately scheduled and well-performed network meetings to create structure in such interaction. We also found that creation of trust in the parent-professional encounter was supported by professionals who were available and provided the parents with adequate information, were skilled at their jobs, and showed empathy and commitment. Therefore, we conclude that a parent-professional interaction that is characterised by structure and trust will provide support and thereby also serve as a holding environment for the parents.

## Figures and Tables

**Table 1.**
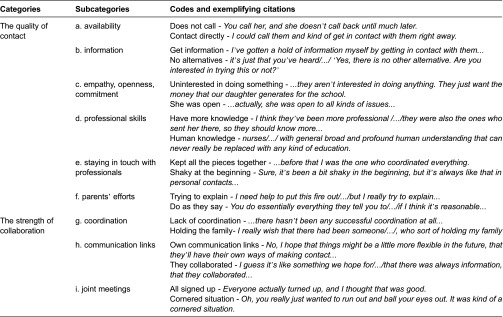
Categories, subcategories and codes
